# Role of SpaO in the assembly of the sorting platform of a *Salmonella* type III secretion system

**DOI:** 10.1371/journal.ppat.1007565

**Published:** 2019-01-22

**Authors:** Maria Lara-Tejero, Zhuan Qin, Bo Hu, Carmen Butan, Jun Liu, Jorge E. Galán

**Affiliations:** 1 Department of Microbial Pathogenesis Yale University School of Medicine, New haven, CT, United States of America; 2 Microbial Science Institute, Yale University School of Medicine, New haven, CT, United States of America; 3 Department of Microbiology and Molecular Genetics McGovern Medical School, The University of Texas Health Science Center at Houston, TX, United States of America; 4 Pathology and Laboratory Medicine, McGovern Medical School, The University of Texas Health Science Center at Houston, TX, United States of America; University of Utah, UNITED STATES

## Abstract

Many bacterial pathogens and symbionts use type III secretion machines to interact with their hosts by injecting bacterial effector proteins into host target cells. A central component of this complex machine is the cytoplasmic sorting platform, which orchestrates the engagement and preparation of type III secreted proteins for their delivery to the needle complex, the substructure of the type III secretion system that mediates their passage through the bacterial envelope. The sorting platform is thought to be a dynamic structure whose components alternate between assembled and disassembled states. However, how this dynamic behavior is controlled is not understood. In *S*. Typhimurium a core component of the sorting platform is SpaO, which is synthesized in two tandemly translated products, a full length (SpaO^L^) and a short form (SpaO^S^) composed of the C-terminal 101 amino acids. Here we show that in the absence of SpaO^S^ the assembly of the needle substructure of the needle complex, which requires a functional sorting platform, can still occur although with reduced efficiency. Consistent with this observation, in the absence of SpaO^S^ secretion of effectors proteins, which requires a fully assembled injectisome, is only slightly compromised. In the absence of SpaO^S^ we detect a significant number of fully assembled needle complexes that are not associated with fully assembled sorting platforms. We also find that although binding of SpaO^L^ to SpaO^S^ can be detected in the absence of other components of the sorting platform, this interaction is not detected in the context of a fully assembled sorting platform suggesting that SpaO^S^ may not be a core structural component of the sorting platform. Consistent with this observation we find that SpaO^S^ and OrgB, a component of the sorting platform, share the same binding surface on SpaO^L^. We conclude that SpaO^S^ regulates the assembly of the sorting platform during type III secretion.

## Author summary

Many pathogenic and symbiotic gram-negative bacteria utilize type III secretion systems to deliver bacterial proteins, known as effectors, directly into the host cell cytosol to promote their survival and the colonization of tissues. Type III secretion systems or injectisomes are large, multiprotein complexes composed of several substructures: the needle complex, a multiring structure with a protruding needle-like appendage anchored in the bacterial envelope; the export apparatus, a set of membrane proteins that form a gate in the inner-membrane for the passage of effector proteins; and the sorting platform, a large cytosolic complex that delivers the effectors to the needle complex in an orderly fashion. In this study, we characterize SpaO, the core component of the *Salmonella* Typhimurium sorting platform. The *spaO* gene encodes two simultaneously translated products, a full length protein (SpaO^L^) and a shorter product (SpaO^S^) encompassing the last 101 aa of the full length product. Here we find that in the absence of SpaO^S^, the sorting platform still forms and functions although slightly less efficiently than in the wild-type situation, and therefore we conclude that SpaO^S^ is most likely not a central structural component of the sorting platform and may play a regulatory role during the cycles of assembly and disassembly that the sorting platform undergoes. In addition, we identify residues critical for the interaction between SpaO^L^ and OrgB and SpaO^L^ and SpaO^S^ and conclude that those interactions might be mutually exclusive further supporting the idea that SpaO^S^ may not be a core structural component of the sorting platform. N-terminal residues in SpaO^L^ are shown to be critical for the formation of the sorting platform. Our findings provide insights into the sorting platform substructure, a highly conserved element in type III secretion systems and may contribute to the development of novel therapeutic avenues to fight infection.

## Introduction

Type III protein secretion systems (T3SSs) are highly specialized multiprotein molecular machines with the capacity to inject bacterially-encoded proteins into target eukaryotic cells. Encoded by a large variety of gram-negative bacteria, T3SSs are central to the interactions of many pathogens and symbionts with their respective hosts[1–3]. The type III secretion machine is made up of several substructures that come together to form the injectisome[1, 4–7].The core component of the injectisome is the needle complex (NC), which is composed of a multi-ring base anchored in the bacterial envelope, and a filament-like extension that protrudes several nanometers from the bacterial surface[4, 6, 8, 9]. The needle filament is traversed by a narrow, ~2 nm channel and is capped at its terminal end by the tip complex, which is thought to be involved in sensing target cells and deploying the translocation pore that mediates the passage of effectors through the target cell plasma membrane[10–15]. The NC is associated to a very large cytoplasmic complex known as the sorting platform, which is responsible for selecting the type III secretion substrates and initiating them into the secretion pathway in the appropriate order[16]. Recent cryo electron tomography (cryo-ET) studies in *Salmonella* Typhimurium and *Shigella flexneri* have provided a high-resolution view of this substructure of the injectisome[5, 17]. The sorting platform exhibits a unique cage-like architecture, enclosed by 6 pods that emerge from the NC and converge into a 6-spoke wheel-like structure that caps it at its cytoplasmic side. In the *S*. Typhimurium T3SS encoded within its pathogenicity island 1 (SPI-1), the cage-like structure is made up of the OrgA, SpaO, and OrgB proteins, which serve as scaffold to place the associated ATPase InvC in close apposition to the export apparatus[5]. The ATPase plays an essential role in initiating substrates in the secretion pathway by removing their associated chaperones and unfolding the effectors prior to their threading through the narrow secretion channel[18].

Several pieces of evidence indicate that, unlike other substructures of the injectisome, the sorting platform exhibits a dynamic behavior and may undergo cycles of assembly and disassembly[19–21]. Fluorescence-imaging studies have shown that cytoplasmically located subunits of the sorting platform can exchange with a pool located in close proximity to the membrane and thus presumably associated to the NC[19, 20]. Consistent with these observations, biochemical and super-resolution imaging studies in live bacteria have shown that a substantial proportion of the core components of the sorting platform are not associated to the NC[21]. Although this dynamic behavior is thought to be essential for the function of the sorting platform, very little is known about its regulation. We show here that SpaO^S^, which is the product of an internally-translated product of *spaO*[22], influences the assembly and/or stability of the sorting platform. Therefore, we propose that SpaO^S^ may regulate the dynamic behavior of this injectisomes substructure.

## Results

### Absence of SpaO destabilizes other components of the sorting platform

**Fig 1:**
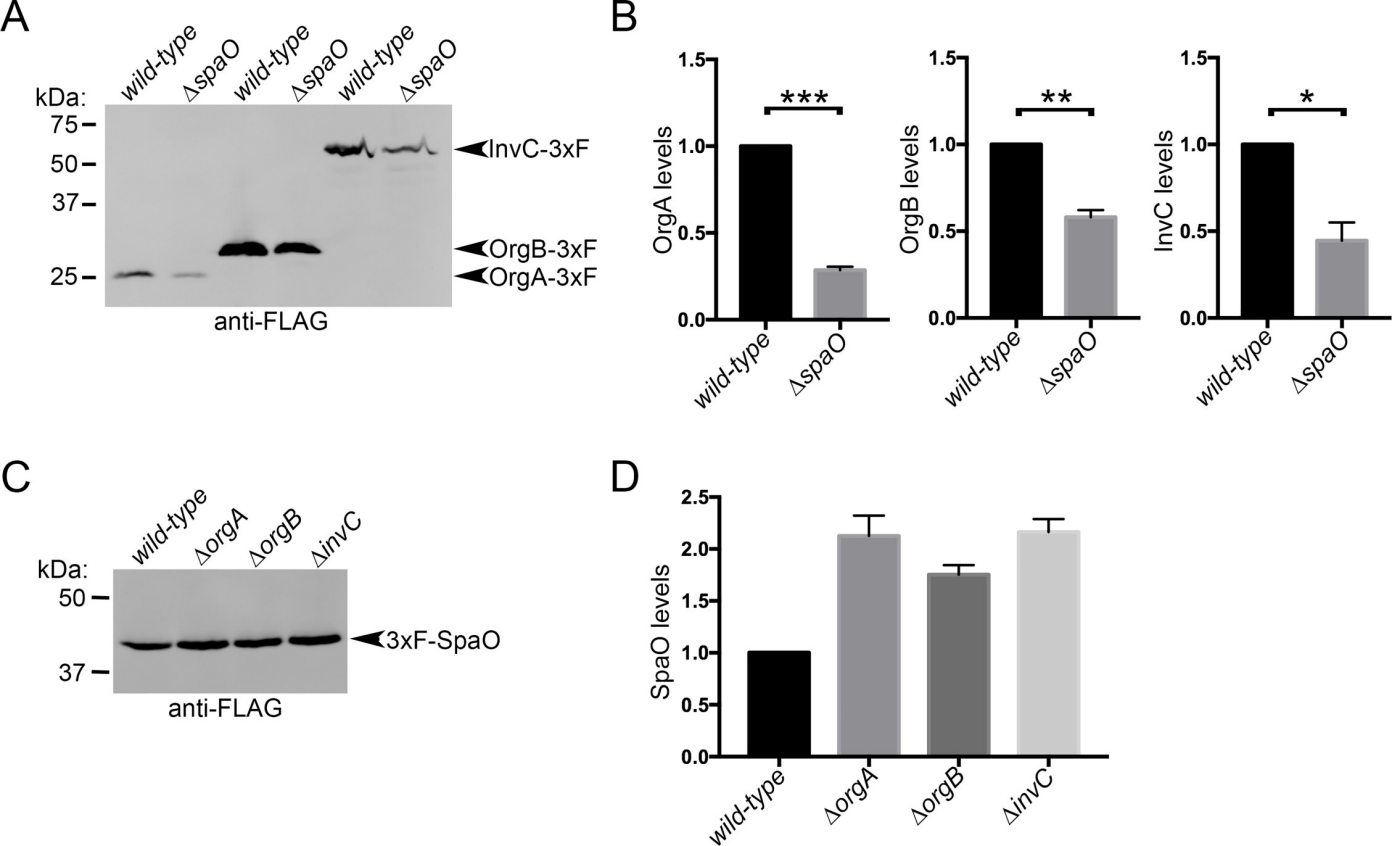
Absence of SpaO destabilizes other components of the sorting platform. (**A**) Anti-FLAG western blot probing the stability of OrgA, OrgB and InvC in the presence or absence of SpaO in whole cell lysates from the indicated *S*. Typhimurium strains. The intensities of the bands were quantified and are shown in (**B**). Values represent the mean ± standard deviation of two independent experiments and levels are expressed relative to the *wild-type* strain (***p = 0.0004, **p = 0.0051, * *p* = 0.0178; unpaired Student’s *t* test). (**C**) Anti-FLAG western blot probing the stability of SpaO in the presence or absence of OrgA, OrgB, or InvC in whole cell lysates from the indicated *S*. Typhimurium strains. The intensities of the bands were quantified and are shown in (**D**). Values represent the mean ± standard deviation of three independent experiments and levels are expressed relative to the *wild-type* strain.

It is often the case that the stability of individual component of multi-protein complexes is affected by the absence of some of the components of such a complex. In addition, insight into the assembly of a multi-protein complex can be often obtained from the observation of the relative stability of its subunits in the presence or absence of one another. We therefore examined the stability of the structural components of the *S*. Typhimurium SPI-1 T3SS sorting platform SpaO, OrgA, OrgB, and InvC in different *S*. Typhimurium mutant strains lacking each one of these components. We found that the levels of OrgA, OrgB, and InvC were significantly reduced in the absence of SpaO (Fig 1A and 1B and Figure A in S1 Text). In contrast, SpaO was stable in the absence of OrgA, OrgB, or InvC (Fig 1C and 1D and Figure A in S1 Text). These observations suggest that SpaO serves as the core component of the sorting platform and therefore it may play a more central role in the coordination of its assembly.

### SpaO^S^ is not essential for T3SS function but is required for the stability of SpaO^L^

**Fig 2:**
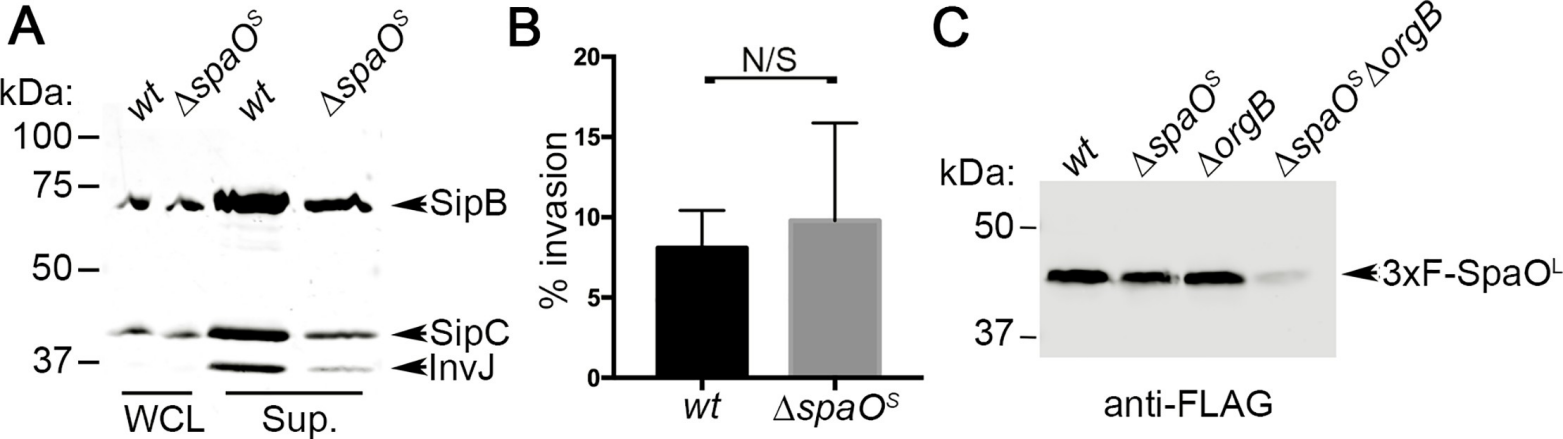
SpaO^S^ is not essential for T3SS function but is required for the stability of SpaO^L^. (**A**) Western blot analyzing SipB, SipC, and InvJ secretion in the presence or absence of SpaO^S^ (WCL = whole cell lysate; Sup. = supernatant) (**B**) Gentamycin protection assay to test the ability of the indicated S. Typhimurium strains in the presence or absence of SpaO^S^ to invade cultured epithelial cells, a measure of the functionality of the SPI-1 T3SS. Numbers represent the percentage of the inoculum that survived antibiotic treatment due to internalization and are the mean ± standard deviation of three independent experiments. The difference between the levels of invasion of the two strains was not statistically significant (*p* = 0.6728, unpaired Student’s *t* test). (**C**) Western blot analyzing the stability of SpaO^L^ in the absence of SpaO^S^, OrgB, or both in whole cell lysates from the indicated *S*. Typhimurium strains.

The internally translated C-terminal polypeptide from the SpaO homologs in *Yersinia* spp. and *Shigella* spp. YscQ^S^ and Spa33^S^, respectively, have been shown to be required for type III secretion in these bacteria[23, 24]. Therefore, it has been proposed that YscQ^S^ and Spa33^S^ are essential structural components of the sorting platform. We investigated the contribution of the internally translated fragment of SpaO (SpaO^S^) to the function of the *S*. Typhimurium SPI-1 T3SS. We constructed a *S*. Typhimurium mutant strain (*S*. Typhimurium *spaO*^*GTG(203)»GCG*^) in which the internal translational initiation GTG codon (codon 203 in the *spaO* gene) for SpaO^S^ was changed to the non-initiating GCG codon, which prevented its expression (Figure B in S1 Text). We then examined the function of the SPI-1 T3SS in a *Salmonella* mutant strain carrying such mutation by biochemical and functional assays. We found that the mutant strain was able to secrete substrates into the culture supernatant although at slightly reduced levels in comparison to wild type (Fig 2A). Furthermore, the mutant was able to enter into cultured epithelial cells, a measure of the function of the SPI-1 T3SS, in a manner indistinguishable from that of wild type indicating that the levels of T3SS-mediated effector secretion, while slightly decreased, were sufficient to mediate a critical function of this system (Fig 2B). These results indicate that, unlike T3SS in other bacteria, SpaO^S^ does not play an essential role in the function of the *S*. Typhimurium SPI-1 T3SS but rather, it increases the efficiency of the secretion system.

It has been previously shown that, similarly to SpaO^S^, its homolog in the *S*. Typhimurium SPI-2-encoded T3SS (SsaQ^S^), is not essential for T3SS function[25]. Instead, it has been proposed that SsaQ^S^ may help the folding of SsaQ^L^ since in the absence SsaQ^S^, the stability of SsaQ^L^ is compromised[25]. However, we found that in the absence of SpaO^S^ the levels of SpaO^L^ were not significantly affected. The observation that SpaO^L^ remains stable in the absence of other component of the sorting platform (see Fig 1C and 1D and Figure A in S1 Text) prompted us to test whether such stability may depend on SpaO^S^. We therefore tested the stability of SpaO^L^ in the absence of both, OrgB and SpaO^S^. We found that the stability of SpaO^L^ is compromised when both SpaO^S^ and OrgB are absent (Fig 2C). These results suggest that SpaO^S^ may serve to stabilize SpaO^L^ prior to its association to other core components of the fully assembled sorting platform.

### SpaO^S^ is required for the efficient assembly of the sorting platform

**Fig 3:**
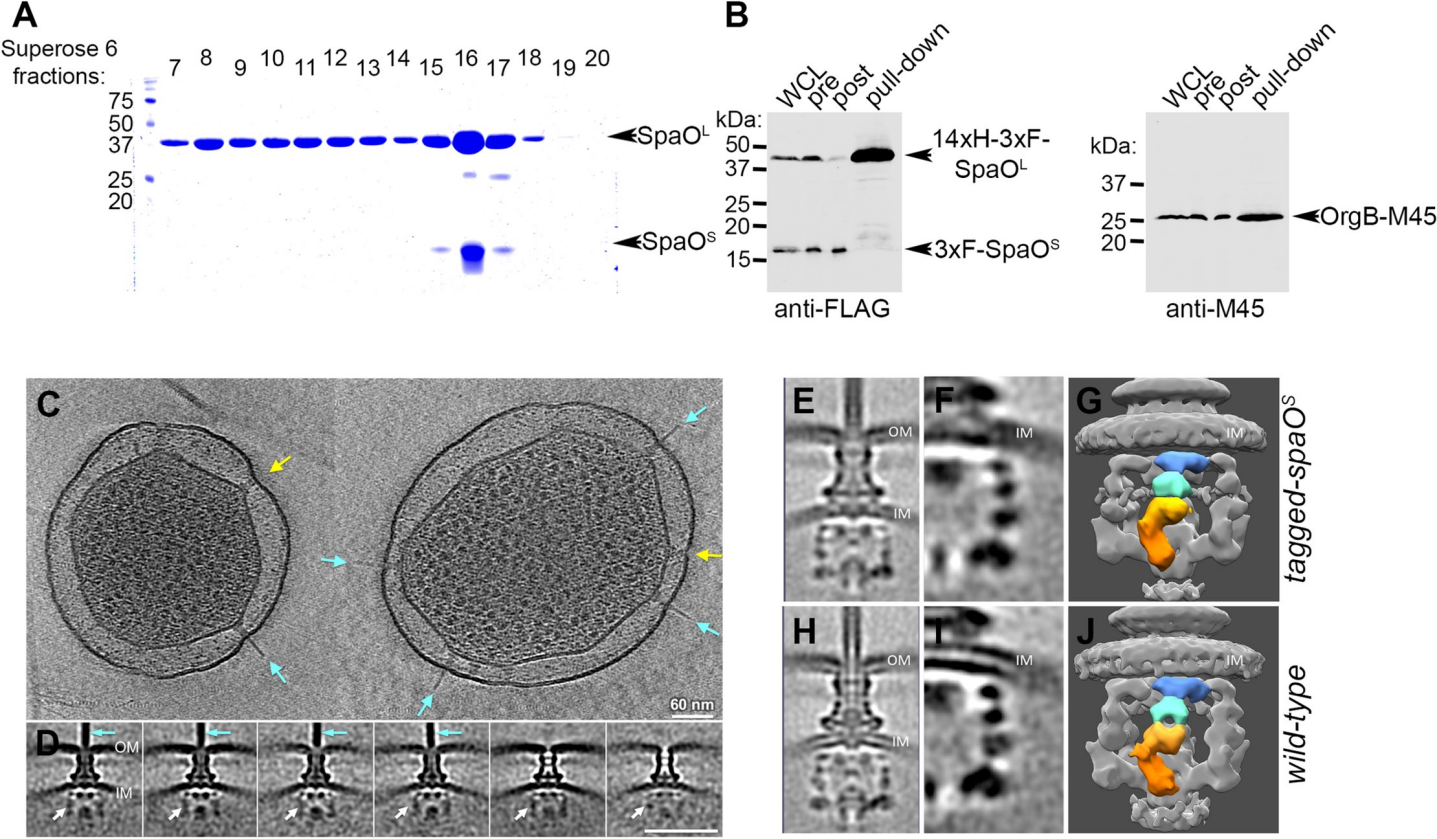
SpaO^S^ is required for the efficient assembly of the sorting platform. (**A**) Superose 6 AKTA fractions of a purified N-terminal His-tagged SpaO, showing the *in-vitro* complex between SpaO^L^ and SpaO^S^. (**B**) Histidine-tag pulldown of SpaO^L^ to probe for its *in-vivo* interactions. FLAG-SpaO^S^ and M45-OrgB in the samples before (pre) and after (post) the pulldowns, or captured in the pulled-down along with SpaO^L^ were detected by Western blotting with either an anti-FLAG (SpaO^S^) or anti-M45 (OrgB) antibody. (**C**) Sections of tomograms from a *∆spaO*^*S*^ *S*. Typhimurium strain. Many of the T3SS injectisomes have the needle substructure (pointing arrow colored cyan), while others do not have the needle (pointing arrow colored in yellow). (**D**) Classification of the injectisomes shows the variable sorting platform. (**E**) An overall structure of the injectisome with a traceable tag on SpaO^S^. A zoom-in section (**F**) and surface view (**G**) show the distinct structure of the sorting platform (**H**) and overall structure of the wild-type injectisome. A zoom-in section (**I**) and surface view (J) of the wild-type injectisome.

We found that, as previously shown[22], *in vitro* SpaO^L^ and SpaO^S^ interact with one another in a 1:2 stoichiometric ratio (Fig 3A). Similar observations have been made with homologs of other T3SSs[23, 24]. However, the investigation of the interaction of SpaO^L^ with SpaO^S^ *in vivo* in the presence of all the T3SS components has not been reported. To determine whether SpaO^S^ interacts with SpaO^L^ *in vivo* we generated a strain that expresses differentially-tagged versions of SpaO^L^ and SpaO^S^ from the *Salmonella* chromosome (Figure C in S1 Text). The interaction between SpaO^L^ and SpaO^S^ was then probed by affinity purification of SpaO^L^ after bacterial growth under conditions that stimulate the expression of all components of the SPI-1 T3SS. No interaction between SpaO^L^ and SpaO^S^ was detected under these conditions even though the interaction of SpaO^L^ and OrgB was readily detected (Fig 3B). These results suggest that the interaction between SpaO^L^ and SpaO^S^ *in vivo* is transient and may not be captured in the presence of other components of the SPI-1 T3SS, conditions that lead to fully assembled sorting platforms. These results also suggest that SpaO^S^ may not be a structural component of the sorting platform.

To further investigate this hypothesis we compared the *in situ* structures of the sorting platforms of wild-type *S*. Typhimurium and that of a mutant unable to produce SpaO^S^ using cryo-ET. We have recently used this approach in combination with bacterial minicells to obtain a high-resolution structure of the entire SPI-1 T3SS injectisome *in situ*[5]. We found that in the absence of SpaO^S^, a significant number of injectisomes displayed a needle filament (Fig 3C). This is consistent with the observation that the injectisome is functional in this mutant strain (Fig 2A and 2B), since assembly of the needle filament requires a fully assembled sorting platform. Sub-tomogram classification was utilized to analyze the sorting platforms present in this mutant strain lacking SpaO^S^. This analysis showed that although there were structures that were indistinguishable from wild type, in the absence of SpaO^S^ there was more heterogeneity in the class averages in comparison to wild type (Fig 3D). Since SpaO^S^ stabilizes SpaO^L^ in the absence of OrgB (Fig 2C), these observations are consistent with a role for SpaO^S^ in the assembly and/or stability of the sorting platform or its components.

It has been previously proposed that homologs of SpaO^S^ may serve as structural components of the fully assembled sorting platform[19]. Although the phenotype of the *∆spaO*^*S*^ mutant is inconsistent with this hypothesis, we probed the potential presence of SpaO^S^ within the assembled sorting platform by adding a traceable density that can be imaged by cryo-ET. We have previously used this approach to map the location of SpaO^L^, OrgA, OrgB and InvC within the assembled sorting platform[5]. We constructed a strain expressing SpaO^S^ tagged at its amino terminus by the fluorescent protein mEos3.2, and examined the sorting platform structures of this strain for the presence of an extra density that could be assigned to SpaO^S^. Comparison of the sorting platform structures from strains expressing tagged (Fig 3E, 3F and 3G) and untagged (Fig 3H, 3I and 3J) versions of SpaO^S^ revealed no detectable additional densities (Fig 3E, 3F and 3G). Although the epitope tagging of SpaO^S^ may affect its function in a manner that cannot be detected by our assays, this observation further supports the hypothesis that SpaO^S^ may not be a structural component of the SPI-1 T3SS sorting platform but rather, may be required for its efficient assembly and/or stability.

### Mutagenesis analysis of SpaO^L^ defines specific functional domains

**Fig 4:**
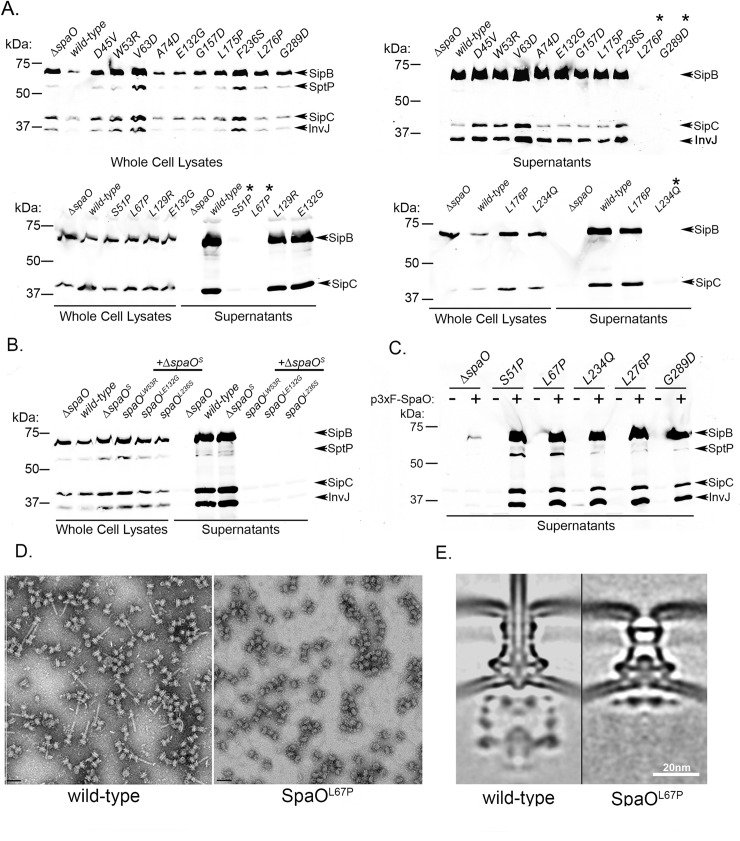
Mutagenesis analysis of SpaO^L^ defines specific functional domains. (**A**) Western blots analyzing SipB, SipC, and InvJ secretion in *S*. typhimurium strains carrying the indicated *spaO* mutations placed in the chromosome. Eleven out of 16 mutants do not show a phenotype in the presence of SpaO^S^. Asterisk symbols denote mutants that showed a secretion phenotype. (**B**) Western blots analysis of SipB, SptP, SipC, and InvJ secretion in *S*. Typhimurium mutant strains expressing the indicated conditional *spaO* mutants placed in the chromosome along with a mutation that abolishes the translation of SpaO^S^. (**C**) Western blots analysis of the secretion of SipB, SipC, and InvJ in S. Typhimurium strains expressing the indicated *spaO* mutants complemented *in-trans* by a *wild-type* copy of *spaO*. (**D**) Electron micrographs of negatively stained needle complexes isolated from wild-type or *spaO*^*L67P*^ *S*. Typhimurium strains show the absence of the needle substructure in the mutant strain. (**E**) Central sections of the cryo-ET sub-tomogram average of the injectisome structure in wild-type and *spaO*^*L67P*^ *S*. Typhimurium strains showing the absence of sorting platform in the strain expressing the mutant allele.

To further characterize the structure and function of SpaO^L^ we carried out a detailed random mutagenesis analysis in an effort to define its functional domains and its protein-interaction network. We used a strategy we have previously described designed to increase the efficiency of the mutagenesis screen[26]. This strategy entails the use of a functional (able to complement a *∆spaO* mutation) chimeric fusion protein between SpaO^L^ and chloramphenicol acetyltransferase (CAT) separated by a flexible linker sequence (Figure D in S1 Text). By imposing the requirement of conferring chloramphenicol resistance, we were able to select against mutations leading to premature termination or gross conformational changes of SpaO^L^ that may prevent the folding of CAT. The *spaO*^*L*^ gene was mutagenized by error-prone PCR and the generated mutants were screened as indicated in the Materials and Methods. We identified 223 loss-of-function mutants out of 12,350 mutants screened. Forty-seven of the identified mutants expressed full-length protein and therefore were analyzed by nucleotide sequencing to determine the location of the mutation(s). Twenty-two of the 47 mutants had a single nucleotide change, which in some instances was independently identified more than once. The identified mutations were distributed throughout the *spaO*^*L*^ coding sequence although clusters of mutations were identified between D45-A74, L129-L134, G157-L176, and L234-G289 (Figure E in S1 Text). Overall, more than 50% of the identified mutations mapped within the amino-terminal half of SpaO, the least characterized of its domains, although mutations were also identified within its carboxy terminal SPOA domains. To examine the effect of the mutations on wild-type *spaO* (i. e. competent to produce both SpaO^L^ and SpaO^S^) we placed sixteen representative mutants mapping to different domains of SpaO in the wild type chromosome and examined their effect on SpaO function. We found that the majority of the mutants tested (11 out of 16) behaved like wild type when placed in the context of a chromosomal wild type *spaO* gene that leads to the synthesis of both SpaO^L^ and SpaO^S^ (Fig 4A). These conditional mutations mapped to the amino-terminal half of SpaO^L^ thus producing wild-type SpaO^S^. These results indicate that the presence of SpaO^S^ was able to suppress the conditional phenotype exhibited by these SpaO^L^ mutants. In the three cases tested, removal of the internal initiating codon of the conditional mutants resulted in the loss-of-function of SpaO thus confirming that these set of mutations were indeed conditional to the absence of SpaO^S^ (Fig 4B). These results suggest that SpaO^S^ may stabilize SpaO^L^ and that the presence of these conditional mutations in SpaO^L^ may enhance the need for the putative chaperone function of SpaO^S^. A similar function has been previously proposed for the SpaO^S^ homolog of the SPI-2 T3SS SsaQ^S^[25].

Five of the 16 mutants exhibited a loss-of-function phenotype even in the presence of wild type SpaO^S^ (Fig 4A). Furthermore, all these mutants were complemented by a wild-type copy of SpaO expressed *in trans*, indicating that they did not exhibit a dominant-negative phenotype (Fig 4C). Three of the identified mutants (SpaO^L234Q^, SpaO^L276P^, and SpaO^G289D^) mapped to the SPOA carboxy terminal domains highlighting the importance of these domains in SpaO function. The SPOA domains have been proposed to be implicated in the formation of higher order SpaO structures through homotypic interactions as well as through interaction with OrgB, another structural component of the sorting platform. However, the specific role of these domains in sorting platform assembly remains poorly understood. Our analysis also identified two loss-of-function mutants (SpaO^S51P^ and SpaO^L67P^) that mapped to the amino terminal third of SpaO (Fig 4A). Unlike the carboxy terminus, little information is available about this domain of SpaO since it has been refractory to structural analysis. To gain insight into the function of the amino terminal domain, we further characterized the phenotype of an *S*. Typhimurium mutant strain expressing SpaO^L67P^. We found that needle complexes purified from this mutant lack the needle substructure (Fig 4D). Since the sorting platform is required for the secretion of the components necessary for needle assembly (i. e. PrgI and PrgJ), these results indicate that this mutant is either unable to assemble the sorting platform or may assemble a non-functional sorting platform. To distinguish between these two possibilities we examined minicells obtained from the *S* Typhimurium *spaO*^*L67P*^ mutant strain by *in situ* cryo-ET. We found that all NC observed lacked the sorting platform indicating that SpaO^L67P^ is unable to direct the assembly of the sorting platform (Fig 4E). These results indicate that the N-terminus of SpaO is essential for the assembly of the sorting platform.

### Mapping the SpaO interaction network through *in vivo* photo cross-linking

**Fig 5:**
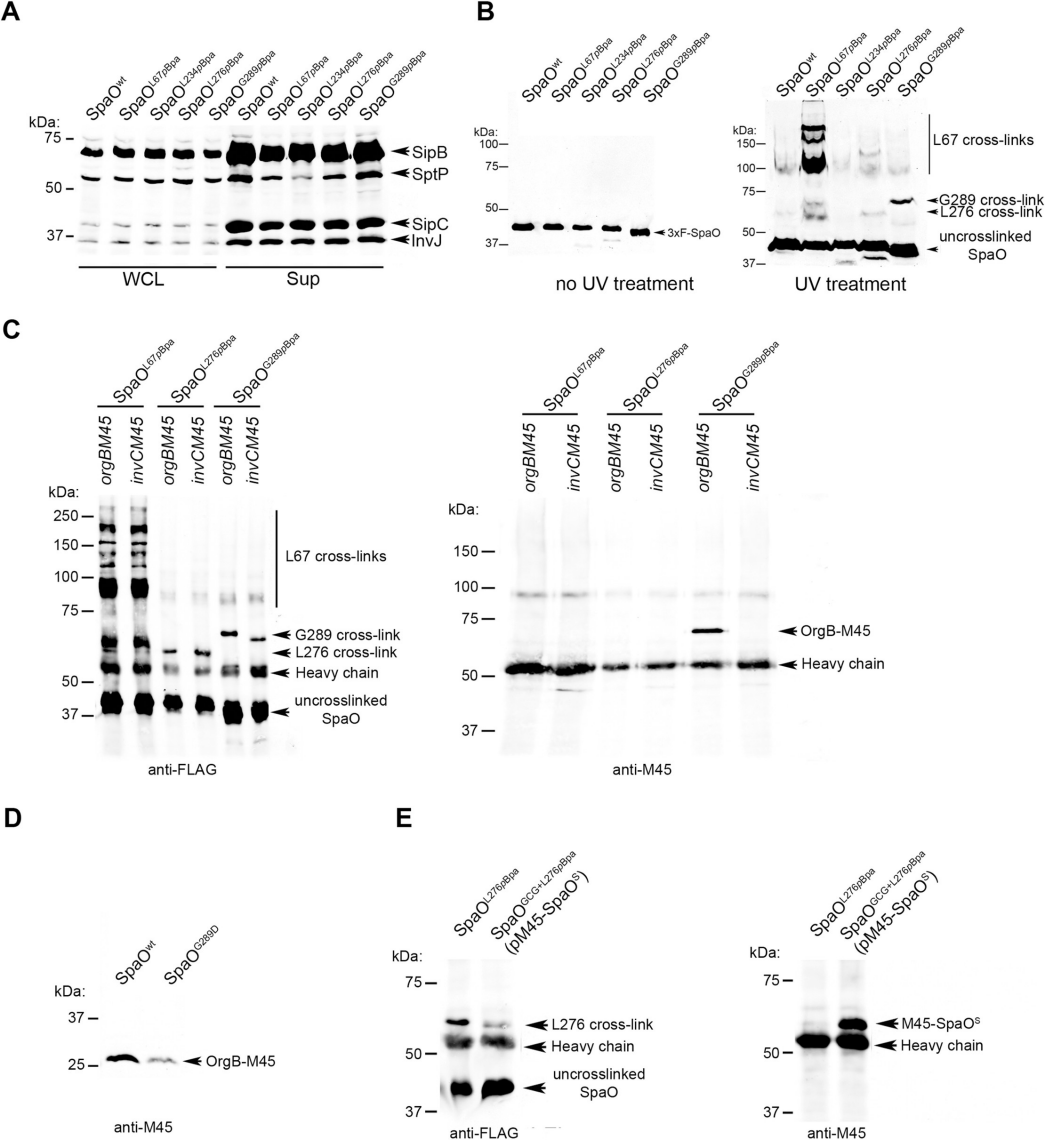
Mapping the SpaO interaction network through *in vivo* photo cross-linking. (**A**) The T3SS of S. Typhimurium strains encoding amber codon *spaO* mutations are functional under amber suppression conditions. Western blots analysis of SipB, SptP, SipC, and InvJ secretion in *S*. Typhimurium strains expressing the indicated *spaO* alleles containing the indicated amber codon mutations. (**B**) SpaO^L67^ mediates homotypic interactions. Western blot analysis of whole cell lysates of *S*. Typhimurium strains encoding the indicated *spaO* amber mutation before and after exposure to UV (as indicated) with an antibody to the FLAG-epitope (present in the different SpaO mutants). The mobility of specific cross-linked species as well as the un-crosslinked SpaO is denoted on the right of the panel. (**C**) SpaO^LG289^ interacts with OrgB. Whole cell lysates of S. Typhimurium strains expressing the indicated FLAG-tagged alleles of SpaO along with M45-epitope tagged versions of InvC or OrgB (as indicated) were exposed to UV (to stimulate the crosslink reaction) and immunoprecipitated with an antibody to the FLAG-epitope. Immunoprecipitates were subsequently probed by western blot with antibodies to the FLAG tag (to detect SpaO, left panel), or to the M45 tag (to detect OrgB or InvC, as indicated, right panel). The mobility of specific cross-linked species as well as the un-crosslinked SpaO is denoted on the right of each panel. (**D**) OrgB is unstable in a *S*. Typhimurium strain expressing SpaO^G289D^. The levels of M45-tagged OrgB in whole cell lysate of wild type *S*. Typhimurium or its isogenic SpaO^G289D^ mutant strain were probed by western blot with an antibody to the M45 epitope. (**E**) L276 in SpaO^L^ interacts with SpaO^S^. Whole cell lysates of S. Typhimuriuum strains expressing FLAG-tagged SpaO^L276pBpa^ along with M45-epitope tagged SpaO^S^ were exposed to UV (to stimulate the crosslink reaction) and immunoprecipitated with an antibody to the FLAG-epitope. Immunoprecipitates were subsequently probed by western blot with antibodies to the FLAG tag (to detect SpaO, left panel), or to the M45 tag (to detect SpaO^S^, right panel). The mobility of specific cross-linked species as well as the un-crosslinked SpaO is denoted on the right of each panel.

Our mutagenesis analysis identified specific SpaO residues required for the assembly of the sorting platform. SpaO^L^ is predicted to engage in multiple interactions with itself as well as with other components of the sorting platform such as OrgA and OrgB. Therefore, it is predicted that some of the identified residues may be involved in some of the defined interactions of this critical component of the sorting platform. To define those potential interaction domains we substituted the codons of the identified critical residues (i. e. SpaO^L67^, SpaO^L234^, SpaO^L276^, and SpaO^G289^) by an amber codon (AUG) so that the unnatural photo-cross-linkable amino acid *p*-benzoyl-L-phenylalanine (*p*Bpa) could be incorporated into SpaO in the presence of an orthogonal aminoacyl tRNA synthetase-tRNA pair[27]. The resulting mutant strains were competent for type III secretion exhibiting a secretion profile in culture supernatants that was indistinguishable from that of the wild-type strain (Fig 5A). These results indicate that SpaO containing the unnatural amino acid *p*Bpa can assemble a wild type sorting platform that is competent for type III secretion function. We then grew the resulting mutant strains under conditions that stimulate the expression of the SPI-1 T3SS and exposed them to UV light to promote site-specific crosslinking. Bacterial cell lysates were then run in SDS-PAGE for western-blot analysis to identify cross-linked species. The UV light treatment resulted in the appearance of distinct cross-linking patterns in lysates from several of the mutant strains (Fig 5B). UV-cross-linking of SpaO^L67*p*Bpa^ resulted in the appearance of a ladder of bands whose mobility suggest that, most likely, represent different multimeric forms of SpaO. Western blot analysis of the bands identified only SpaO (Fig 5C), which is consistent with the conclusion that these bands represent crosslinks of SpaO to itself as a consequence of its multimerization. The molecular weight of the cross-linked bands suggests that SpaO may form a tetrameric complex. This is consistent with the predicted stoichiometry of SpaO, 24 copies[21], and its organization in the assembled 6-pod sorting platform[5]. These results also indicate that the amino terminal domain of SpaO plays a critical role in the formation of high order homotypic structures, which are essential for the assembly of the sorting platform.

UV crosslinking of *S*. Typhimurium SpaO^L276pBpa^ and SpaO^G289pBpa^ cells also resulted in the detection of distinct cross-linked species (Fig 5B and 5C). To identify the cross-linked proteins we probed the cross-linked bacterial cell lysates for the presence other components of the sorting platform functionally tagged with a different (M45) epitope tag. Through this analysis we found that SpaO^G289Bpa^ cross-linked to OrgB indicating that G289 is required for SpaO interaction with OrgB (Fig 5C). We found that the stability of OrgB is compromised in the absence of SpaO (see Fig 1). Therefore, we reasoned that if OrgB is unable to interact with SpaO^G289D^ its stability should be compromised in the presence of this mutation. Consistent with this hypothesis, the amount of OrgB in cell lysates of a S. Typhimurium *spaO*^*G289*D^ mutant was reduced (Fig 5D) further supporting the conclusion that OrgB engages in critical interactions with the G289 residue in SpaO.

The observed SpaO^L276*p*Bpa^ cross-linked band was smaller in size than that of the SpaO^G289*p*Bpa^-OrgB cross-link but consistent with a band corresponding to a SpaO^L^-SpaO^S^ cross-link. To test this hypothesis we expressed M45-epitope-tagged SpaO^S^ in a *S*. Typhimurium strain expressing SpaO^L276*p*Bpa^ and unable to translate SpaO^S^. Bacterial cell lysates after exposure to UV light were then analyzed by western blotting. This analysis indicated that the cross-linked band was composed of SpaO^L276*p*Bpa^-SpaO^S^ (Fig 5E) indicating that L276 is essential for the formation of a heterodimer between the SPOA2 domains of SpaO^L^ and SpaO^S^. Since the N-terminus of OrgB has been shown to interact with a SPOA1-SPOA2 heterodimer of SpaO^L^ making extensive contacts with both SPOA domains[22], these results indicate that the binding of SpaO^L^ to OrgB and SpaO^S^ is mutually exclusive, which is consistent with our inability to detect an interaction between SpaO^L^ and SpaO^S^ in *S*. Typhimurium in the presence of other T3SS components. These observations are also consistent with our conclusions that SpaO^S^ may not be a structural component of the sorting platform but that it plays a role in the course of its assembly and/or dynamic behavior during type III secretion.

## Discussion

The sorting platform is an essential substructure of the T3SS injectisome, which is critical for the recruitment and sorting of protein substrates destined to travel the type III secretion pathway[16]. Recent cryo-ET studies have shown, in *S*. Typhimurium, that the sorting platform is made of a multi-protein cage-like scaffold, integrated by OrgA, SpaO, and OrgB, which serves as support for another component of this substructure, the ATPase InvC, which is involved in the initiation of substrates into the secretion pathway[5]. Unlike other substructures of the T3SS injectisome, the sorting platform is thought to exhibit a behavior that may involve cycles of assembly and disassembly[19, 20]. However, very little information is available about the mechanisms and functional significance of this dynamic behavior. Furthermore, little is known about the mechanisms of assembly of this critical component of the T3SS machine. In this paper we have used a multidisciplinary approach to gain insight into the role of SpaO in the assembly of the sorting platform.

We found that in the absence of SpaO, other components of the sorting platform are destabilized. In contrast, the absence of other sorting platform components did not destabilize SpaO indicating that it must play a central role in its assembly. Consistent with this role, cryo-ET studies have mapped the location of SpaO to the central region of the pods that form the core of the cage-like structure, a position that would allow it to interact with the other components of the sorting platform[5]. In fact, our mutagenesis and cross-linking studies identified critical domains of SpaO involved in interactions with other components of the sorting platform. As previously demonstrated[22], we found that the carboxy terminal SPOA domains of SpaO mediate its interaction with OrgB, which caps the cage-like structure at its cytoplasmic side serving as a cradle for InvC. In addition, our genetic screen identified critical residues at its poorly characterized amino terminus, which are essential for the oligomerization of SpaO, and presumably, for its interaction with other components of the sorting platform. We hypothesize that this interaction network allows SpaO to nucleate the assembly of the sorting platform. Consistent with this hypothesis, we have shown by cryo-ET *in situ* analysis that a single amino acid substitution in SpaO that disrupts this interaction network resulted in the complete absence of the sorting platform.

*spaO* and its homologues are unusual in that they are translated as two products, a full length product (SpaO^L^) and a shorter version composed of their carboxy terminal third (SpaO^S^), the product of an internal translational initiation site[23–25]. The precise role of SpaO^S^ is unclear and the available evidence suggests that its function may differ in different T3SSs. For example, in the case of the *Yersinia* T3SS, it has been reported that the SpaO^S^ homolog (YscQ^S^) is essential for type III secretion[23]. Based on this observation, it has been suggested that YscQ^S^ is a structural component of the sorting platform. However, we found that the absence of SpaO^S^ resulted in a rather mild secretion phenotype and no detectable functional phenotype, observations that are inconsistent with a crucial structural role for this component of the sorting platform. Similar observations have been made in the case of the *S*. Typhimurium SPI-2 T3SS homolog SsaQ^S^[25]. If not a structural component of the sorting platform, what could be the function of SpaO^S^? We hypothesize that SpaO^S^ may play a regulatory role during assembly and disassembly of the sorting platform. We based our hypothesis on the following observations. First, the mild phenotype of its absence, which is more consistent with a regulatory rather than a structural role. Second, the observation that in the absence of SpaO^S^ we detect needle complexes that while displaying a needle filament *in situ*, lack a fully assembled sorting platform. Since assembly of the needle filament requires a fully functional sorting platform, we take this observation to mean that those needle complexes must have been previously associated with a sorting platform but the absence of SpaO^S^ may impede or delay its efficient re-assembly. Third, the stability of SpaO^L^ in the absence of other components of the sorting platform requires SpaO^S^. This observation suggests that prior to the assembly of the sorting platform (or during the cycles of assembly and disassembly), SpaO^S^ may play a crucial role in chaperoning SpaO^L^. Fourth, at least in the fraction of *in situ* structures that we were able to visualize, we detected no obvious differences in the structure of the fully assembled injectisomes from the wild-type and the *∆spaO*^*S*^ mutant strains. Finally, we were not able to detect the presence of SpaO^S^ in fully assembled injectisomes. Taken together, these data suggest a regulatory rather than a core structural role for SpaO^S^ in the assembly of the sorting platform. What this role might be is unclear but stabilizing SpaO^L^ when not associated to other components of the sorting platform must be a central element of this function.

We have shown here that the two forms of SpaO, SpaO^L^ and SpaO^S^, play a central role in the assembly of the *S*. Typhimurium T3SS sorting platform. The conserved nature of the T3SS components suggests that this function may be maintained in other T3SS.

## Materials and methods

### Bacterial strains, plasmids and growth conditions

All the strains used in this study are derivatives of *Salmonella enterica* Typhimurium SL1344. Genetic modifications were introduced in SL1344 by allelic exchange using R6K suicide vectors[28] in *E*. *coli ß2163 ∆nic35* as donors of the modified allele[29]. All plasmids in this study were constructed using Gibson assembly [30]. The resulting modified strains were screened by PCR and confirmed by western blot and/or functional assays to behave in a manner indistinguishable from wild type. A complete list of plasmids and strains used in this study can be found in Table S1.

To prevent recombination between the upstream *spaO*^*LGTG(203)>GCG*^ allele and the downstream 3xFlag-tagged *spaO*^*S*^ in the strain schematically presented in Figure C in S1 Text the coding sequence of *spaO*^*S*^ was changed to:

ATGGACTACAAAGACCATGACGGTGATTATAAAGATCATGACATCGATTACAAGGATGACGATGAGACCCTGGATATCCAGCATATTGAAGAGGAGAACAACACGACCGAAACCGCGGAAACCCTGCCGGGCCTGAACCAGTTACCGGTGAAACTGGAATTCGTCTTATATCGCAAAAATGTCACGCTTGCGGAACTTGAAGCGATGGGTCAACAGCAACTCTTGTCGTTACCGACGAACGCAGAGTTAAATGTCGAGATCATGGCCAACGGCGTCCTTTTAGGCAACGGTGAGTTAGTGCAAATGAACGATACGCTGGGTGTCGAAATTCACGAGTGGTTATCGGAAAGCGGGAACGGTGAGTGA

The underlined sequence corresponds to the 3xFlag-tag sequence. The changes did not alter the actual amino acid sequence of SpaO^S^. The new sequence was generated as a synthetic minigene by IDT, Integrated DNA technologies, Inc.

Strains were typically maintained in Luria broth (LB). To induce optimal expression of the SPI-1 T3SS, LB was modified to contain 0.3 M NaCl and cultures were grown under low aeration to an OD_600_ of ~0.9[31]. When appropriate, further induction of SPI-1 was achieved by expression of *hilA*[32], the master regulator of SPI-1 T3SS expression, from an arabinose inducible plasmid.

### Secretion assay

Overnight cultures of the strains of interest were grown in LB with the appropriate antibiotics. The O/N cultures were diluted 1:20 in 10 ml of 0.3 M NaCl LB containing the appropriate antibiotics and 0.05% arabinose when the *hilA* plasmid was present. The subcultures were grown under low aeration conditions until an OD_600_ of ~ 0.9 (4 to 5 hours). Then, 1 ml of the subculture was transferred to a 1.5 ml microfuge tube, the cells were pelleted at maximum speed for 1 min, resuspended in 100 μl of SDS-PAGE loading buffer and saved as the whole cell lysate (WCL) control at 10x concentration. The rest of the culture was spun down and the supernatant was filtered using a 0.45 μm syringe filter to remove remaining bacterial cells. Proteins in the supernatant were recovered by trichloroacetic acid (TCA) precipitation, and the protein pellet was resuspended in 100 μl of SDS-PAGE loading buffer, resulting in the sup sample at 100x concentration.

### Western blot imaging and quantification

All Western blots were imaged using the Odyssey Li-Cor system (LI-COR Biosciences) and near-infrared secondary fluorescence antibodies that capture data over the entire linear range in a single image. Quantification of the Western blots was performed using Image Studio Lite software specifically desing by Li-Cor for Western blot quantification.

### Gentamicin protection assay

Int 407 embryonic intestinal epithelial cells, seeded in a 24-well plate, were infected in triplicates with the wild-type or *∆spaO*^*S*^ *S*. Typhimurium strains grown under SPI-1-T3SS expression-inducing conditions with a multiplicity of infection (m. o. i.) of 10 in Hank’s balanced salt solution with calcium and magnesium (Gibco 14025092). Dilutions were plated to determine the exact inoculum used for each strain. Infection was let to proceed for 30 min, after which the cells were washed 3x with 0.5 ml of PBS, and incubated for 1 h in the presence of DMEM containing 10% BCS and 50 μg/ml of gentamicin to kill extracellular bacteria. Cells were washed 3x with 0.5 ml of PBS, lysed in 0.5 ml of PBS + 0.05% Na-deoxycholate, and the number of colony forming unites (c. f. u.) was determined by dilution plating in LB agar plates containing 100μg/ml of streptomycin. Bacterial invasion was expressed as the percentage of the inoculum surviving the gentamicin treatment.

### Purification of His-tagged SpaO

A 50 ml overnight culture of *E*. *coli* Lemo21(DE3) (New England Biolabs C2528J) harboring plasmid pSB3775 in LB containing 50 μg/ml of kanamycin and 30 μg/ml of chloramphenicol was diluted in 1 L of LB containing the same antibiotics. The culture was grown at 37°C and 220 rpm to an OD_600_ of 0.6 and then induced with 0.5 mM IPTG for 5 hours. Cells were pelleted at 6,000 rpm and the pellet resuspended in 10 ml of lysis buffer (50 mM NaH_2_PO_4_, 300 mM NaCl, 10 mM imidazole, 1mM Mg_2_Cl, 2.5U of DNAse, and cOmplete™ Protease Inhibitor Cocktail [Sigma 11697498001]). Cells were lysed using the One Shot cell disrupter (Constant Systems Ltd., Northants, UK). After lysis, cellular debris was removed by centrifugation, and the cleared supernatant was transferred to a fresh tube. Two hundred microliters of Ni-NTA agarose resin (Qiagen 30210) were added to the lysate, and after 3 h incubation at 4°C under rocking conditions, the lysate containing the agarose resin was applied to an empty chromatography column (Bio-Rad 7311550). The beads were washed three times with 10 ml of lysis buffer containing 20 mM imidazole and the bound protein was eluted in five 1 ml aliquots of lysis buffer containing 250 mM imidazole. The protein concentration was estimated using the Bio-Rad Protein Assay (Bio-Rad 500–0006) and fractions containing high amounts of protein were concentrated prior to loading into a Superose 6 10/300 GL (GE Life Sciences 17517201) column using an ÄKTA purifier system (GE Life Sciences). One ml fractions were collected, and 20 μl of each fraction were subsequently analyzed by SDS-PAGE and Coomassie Brilliant Blue R-250 (ThermoFisher 20278) staining.

### Pull-down assay

Overnight cultures of *S*. Typhimurium strains expressing the indicated his-tagged proteins and carrying a plasmid encoding *hilA* expressed from an arabinose-inducible promoter[32, 33] were diluted 1:20 in flasks containing 150 ml of LB containing 0.3 M NaCl, 100 μg/ml ampicillin, and 0.05% arabinose. Cultures were grown at 37°C under 100 rpm shaking (low aeration) conditions to an OD_600_ ~ 0.6, cells were pelleted at 7,000 rpm, resuspended in 2.5 ml of PBS containing 15 mM imidazole, cOmplete EDTA-free protease inhibitor cocktail (Sigma 4693159001), and lysed using a One Shot table top homogenizer (Constant Systems Ltd, Northants, UK). Debris was removed by centrifugation and the cleared lysate was transferred to a fresh 2 ml microcentrifuge tube. One hundred μl of Ni-NTA agarose (Qiagen 30310) was added to each sample and the tubes were incubated for 1 hr at 4°C under rocking conditions. After binding, beads were washed 4x with 1 ml of PBS containing 20 mM imidazole. Bound protein was eluted in 100 μl of PBS containing 250 mM imidazole or by boiling the beads in SDS-PAGE running buffer. Samples were collected throughout the procedure and analyzed by western blot.

### Isolation of minicells

Minicell producing bacterial strains were grown overnight at 37°C in LB containing 0.3M NaCl. Fresh cultures were prepared from a 1:100 dilution of the overnight culture and then grown at 37°C to late log phase in the presence of ampicillin (200 μg/mL) and L-arabinose (0.1%) to induce the expression of regulatory protein HilA and thus increase the number of injectisomes partitioning to the minicells[34]. To enrich for minicells, the culture was centrifuged at 1,000 x g for 5 min to remove bacterial cells, and the supernatant fraction was further centrifuged at 20,000 x g for 20 min to collect the minicells. The minicell pellet was resuspended in PBS and mixed with 10 nm colloidal gold particles and deposited onto freshly glow-discharged, holey carbon grids for 1 min. The grids were blotted with filter paper and rapidly frozen in liquid ethane, using a gravity-driven plunger apparatus as described previously[5, 17].

### Cryo-electron tomography data collection and reconstruction

The frozen-hydrated specimens were imaged with 300kV electron microscopes. The tomographic package SerialEM [35] was utilized to collect single-axis tilt series from -51° to +51° using Polara or Titan electron microscopes equipped with a field emission gun and a direct detection device (Gatan K2 Summit). The tilt series was aligned and reconstructed using IMOD [36]. In total, 2,452 tomograms (3,600 × 3,600 × 400 pixels) were generated for detailed examination of the sorting platform in several mutants (Table S2).

### Sub-tomogram analysis

Sub-tomogram analysis was accomplished as described previously [17] to analyze the injectisomes. Briefly, we first visually identified the injectisomes on each minicell. Two coordinates along the needle were used to estimate the initial orientation of each particle assembly. For initial analysis, 4 × 4 × 4 binned sub-tomograms (128 × 128 × 128 voxels) of the intact injectisome were used for alignment and averaging by using the tomographic package I3 [37, 38]. Then multivariate statistical analysis and hierarchical ascendant classification were used to analyze the needle tip complex [38].

### 3-D visualization

IMOD and UCSF Chimera [39] were used to visualize the sub-tomogram average structures of the T3SS injectisome.

### Error prone PCR mutagenesis

The mutagenesis procedure was based on a previously described strategy [26], which relies on the use of a chimeric fusion protein between the protein to be mutagenized (SpaO^L^, *spaO*^GTG203TTG^, in this case) and chloramphenicol acetyltransferase (CAT) separated by a flexible linker sequence. By imposing the requirement of chloramphenicol resistance, mutations leading to premature termination or gross conformational changes can be counter-selected. A detailed schematic representation of the plasmid employed for the mutagenesis (pSB4545) can be found in Figure D in S1 Text. This plasmid was able to complement the secretion phenotype of a *S*. Typhimurium *∆spaO* mutant strain (Figure D in S1 Text). The mutagenic PCR was performed as described [40], but without the addition of MnCl_2_ to reduce the mutation frequency. SpaO^L^ was amplified under mutagenic conditions using forward (ATGGACTACAAAGACCATGACGG) and reverse (TTCTCTCTAGAAGGCAGGTGTCCCTGCAC) primers and ligated to plasmid pSB4545. The ligation was transformed into *E*. *coli* selecting for kanamycin, colonies were pooled and plasmid DNA was extracted and electroporated into a *∆spaO S*. Typhimurium strain. During the set-up process, we found that the SpaO-CAT fusion protein required time to fold and confer chloramphenicol resistance and that plating the electroporation directly into chloramphenicol-containing plates yielded no colonies. For this reason, the electroporated *∆spaO S*. Typhimurium was plated on 30 μg/ml kanamycin LB plates (to select for the plasmid) and kanamycin resistant colonies were then replicated onto chloramphenicol-containing plates (10 μg/ml). Colonies that were chloramphenicol-resistant were then assayed for type III secretion function by a dot-blot assay.

### Dot-blot assay to screen loss-of-function mutants

Chloramphenicol resistant *S*. Typhimurium expressing M45 epitope tagged SopB and harboring the mutagenized plasmid were inoculated in a 96-well plate containing 200 μl of 0.3M NaCl LB and 10 μg/ml of chloramphenicol per well, and incubated O/N at 37°C with gentle shaking. Using a 96 pin replicator (Boekel Scientific 140500) a small volume of culture was transferred to a nitrocellulose membrane, which was let to dry and then fixed by exposure to chloroform vapors. This procedure did not permeabilize the bacterial cells. The presence (secretion positive) or absence of SopB (secretion negative) on the bacterial surface was then probed by treating the membranes with a monoclonal antibody to the M45 epitope following standard procedures (Figure F in S1 Text). The clones that showed a loss-of-function phenotype in the dot-blot assay were then analyzed by western blot to examine the expression of the full length SpaO-CAT chimeric protein (Figure G in S1 Text). Clones that showed a loss-of-function phenotype and expressed the full length SpaO-CAT protein were then sequenced to determine the location of the mutation(s) (Figure E in S1 Text). Following the outlined screen, 12,350 kanamycin-resistant clones were picked and replicated on chloramphenicol plates. Of those clones, 1,965 clones (17.37%) were chloramphenicol resistant and were then assayed for type III secretion function on dot-blot assays; 223 of the assayed clones were selected as loss-of-function and checked to confirm that they express the full length SpaO-CAT fusion protein. Forty seven clones passed all the requirements and were sequenced. Twenty-two of the 47 clones had a single mutation, some of which were identified independently more than once. Figure E in S1 Text. shows the location of the single mutations identified. A summary of the mutagenesis results can be found in Table S3.

### Site-specific *in vivo* photo-crosslinking

For site-specific in vivo photo-crosslinking, the photoreactive unnatural amino acid *p*Bpa[41] was incorporated into SpaO by replacing the codon of the targeted amino acid with a TAG amber codon. Incorporation of *p*Bpa was accomplished by amber codon suppression provided by the presence of plasmid pSUP encoding an *E*. *coli* nonsense suppressor tRNA-tRNA synthetase system that can recognize and incorporate *p*Bpa at the TAG amber codon[27]. Overnight cultures of the strains encoding the different TAG-containing *spaO* alleles, and carrying plasmids encoding the suppression system and *hilA* expressed under an arabinose-inducible promoter were diluted 1:20 in 10 ml of 0.3M NaCl LB containing 100 μg/ml ampicillin, 10 μg/ml chloramphenicol, 0.1% arabinose, and 1mM *p*Bpa and grown at 37°C to an OD_600 of_ ~ 0.9. Bacterial cells were pelleted and resuspended in 5 ml of PBS. Half of the resuspended culture was placed in a 60 mm tissue culture dish and irradiated at 365 nm with a hand-held UV lamp and the other half was left unexposed to the UV lamp. Cells were then pelleted and resuspended in 250 μl of SDS-PAGE loading buffer (20x concentration) and 20 μl of the UV-treated or control samples were loaded onto a SDS-PAGE gel for western blot analysis. In some instance the site-specific in vivo crosslinking experiments were scaled up (up to 200 ml of culture) and after crosslinking the crosslinked species were concentrated by anti-flag immunoprecipitation (see section below).

### Immunoprecipitation of crosslinked proteins

After UV-crosslinking as indicated above, bacterial cells were pelleted and resuspended in 3 ml of TBS containing 1mM MgCl_2_ and cOmplete EDTA-free protease inhibitor cocktail (Sigma 4693159001). The suspended cells were lysed by sonication (5 minutes @ 35% amplitude with cycles of 3 sec ON and 7 sec OFF). Debris was removed by centrifugation, the clarified lysate was transferred to a fresh tube and 50 μl of anti-Flag M2 affinity gel (Sigma A2220) were added. Samples were incubated for ~4 hrs at 4°C under rocking conditions, beads were washed 4x with 1 ml of TBS containing 0.05% Tween-20, resuspended in 50 μl of SDS-PAGE running buffer, and boiled to elute all bound proteins.

### Needle complex purification and EM imaging

The needle complex purification was carried out by maltose-binding protein (MBP) affinity purification as follows. An MBP-tagged PrgH allele was introduced into an *S*. Typhimurium strain carrying the SpaO^L67P^ mutation. Two liters of 0.3M NaCl containing 100 μg/ml of ampicillin and 0.1% arabinose were inoculated with the strains of interest and grown for ~10 hrs under gentle (100 rpm) shaking. Cells were recovered by centrifugation, resuspended in 10 ml of lysis buffer (200mM Tris pH 7.5, 20% sucrose, 1mM EDTA, 0.25mg/ml of lysozyme and cOmplete EDTA-free protease inhibitor cocktail [Sigma 4693159001]) and incubated on ice for 1 hr. Cells were incubated for 5 min at 37°C and lysed by the addition of 0.5% N-Dodecyl- *ß-*D-maltoside (DDM) (Anatrace D310S). Cells were incubated at 37°C for additional 5 to 10 min while monitoring lysis. Cells were then transferred to ice and further incubated for an additional hour. Debris was removed by centrifugation and the clarified lysate was transferred to a fresh tube. Two hundred microliters of amylose resin were added and the suspension was incubated O/N at 4°C under rocking conditions. Beads were then washed 4x with 10 ml of washing buffer (20 mM Tris pH 7.5, 100 mM NaCl, 1 mM EDTA) and finally resuspended in 50 μl of washing buffer containing 20 mM of maltose. After 1 hr incubation on ice with occasional tapping, beads were removed by centrifugation and the needle complex containing supernatant was transferred to a fresh tube. Samples (3.5 μl) were directly applied onto glow-discharged grids bearing a continuous carbon film (EMS CF300-Cu). After two minutes, the sample was blotted, then stained with 2% (w/v) uranyl acetate. Images were recorded on a FEI Tecnai-12 electron microscope (LaB6, 120KV) equipped with a 4096x4096 pixel Gatan Ultrascan CCD camera.

## Supporting information

S1 Text **Figure A.** (A) Quantification of OrgA, OrgB, and InvC levels in the absence of SpaO, duplicate biological experiments loaded on the same gel. (B) Quantification of SpaO levels in the absence of OrgA, OrgB or InvC. Biological triplicates loaded on the same gel. In both cases, loading levels were normalized based on the quantification of a protein whose levels were constant as observed by Coomassie Brilliant Blue (CBB) staining (gel underneath Western blot). Quantifications were performed using the Image Studio Lite software from LI-COR Biosciences.
**Figure B.** SpaO^S^ is translated from the internal codon 203 in the *spaO* gene. (A) Whole cell lysates of either the *wild-type* strain (*spaO-3xF*) or a strain where the initiating codon for SpaOS is mutated (*spaOGTG(203)>GCG-3xF*) where run on a 15% SDS-PAGE gel and blotted against the 3xF-tag to detect SpaO^L^ and SpaO^S^. **Figure C.** Schematic representation of the *spaO* locus in the *S*. Typhimurium strain SB3137. The coding sequence of SpaO^S^ was modified without altering the protein sequence to minimize recombination between *spaO*^*L*^ and downstream *spaO*^*S*^. **Figure D.** (**A**) Schematic representation of plasmid pSB4545 used in the mutagenesis experiments. The plasmid expresses SpaO^L^ fused to a chloramphenicol acetyl transferase (cat) gene and carries a point mutation in *spaO* (the GTG codon at aa 203 changed to a GCG) to prevent the translation of SpaO^S^. The SpaOL open reading frame in this plasmid was mutagenized by error-prone PCR as described in Materials and Methods. (**B**) Complementation of a *S*. Typhimurium *∆spaO* mutant (expressing the M45-tagged effector protein SopB) by the plasmid pSB4545 (pMut). Whole cell lysates (WCL) and culture supernatant proteins (Sup) were separated in a 10% SDS-PAGE gel and blotted against M45 tag to detect SopB. **Figure E.** Unique point mutations identified by error prone PCR depicted on the SpaO protein sequence. The start of SpaO^S^ is indicated in red. **Figure F.** Example of dot blots used to screen for loss-of-function SpaO mutants. The dot blots showed the detection of the SPI-1 T3SS effector SopB in the supernatant of *S*. Typhimurium *∆spaO* mutant complemented with the pSB4545 plasmid expressing SpaO^L^ fused to cat as positive control (+), the vector alone (-), or different SpaO^L^ mutants generated by error prone PCR as described in the Materials and Methods section.Controls are circled in blue, while putative type III secretion mutants are circled in red. **Figure G.** An example of a western blot analysis of whole cell lysates of *S*. Typhimurium expressing different FLAG-epitope-tagged SpaO^GTG(203)>GCG^-cat fusion proteins to examine the stability of different mutants generated by error prone PCR and selected as type III secretion defective by the dot blot presented in Supplementary Figure S5. Only those mutants that expressed the full-length fusion protein (indicated by an asterisk *) were analyzed further.
(DOCX)
