# Supplementary material for: Role of SpaO in the assembly of the sorting platform of a Salmonella type III secretion system
Source: PLoS Pathog. 2019 Jan 22;15(1):e1007565. doi: 10.1371/journal.ppat.1007565 (PMC6358110; doi:10.1371/journal.ppat.1007565)
Supplement: S1 Text — Figure A. (A) Quantification of OrgA, OrgB, and InvC levels in the absence of SpaO, duplicate biological experiments loaded on the same gel. (B) Quantification of SpaO levels in the absence of OrgA, OrgB or InvC. Biological triplicates loaded on the same gel. In both cases, loading levels were normalized based on the quantification of a protein whose levels were constant as observed by Coomassie Brilliant Blue (CBB) staining (gel underneath Western blot). Quantifications were performed using the Image Studio Lite software from LI-COR Biosciences. Figure B. SpaOS is translated from the internal codon 203 in the spaO gene. (A) Whole cell lysates of either the wild-type strain (spaO-3xF) or a strain where the initiating codon for SpaOS is mutated (spaOGTG(203)>GCG-3xF) where run on a 15% SDS-PAGE gel and blotted against the 3xF-tag to detect SpaOL and SpaOS. Figure C. Schematic representation of the spaO locus in the S. Typhimurium strain SB3137. The coding sequence of SpaOS was modified without altering the protein sequence to minimize recombination between spaOL and downstream spaOS. Figure D. (A) Schematic representation of plasmid pSB4545 used in the mutagenesis experiments. The plasmid expresses SpaOL fused to a chloramphenicol acetyl transferase (cat) gene and carries a point mutation in spaO (the GTG codon at aa 203 changed to a GCG) to prevent the translation of SpaOS. The SpaOL open reading frame in this plasmid was mutagenized by error-prone PCR as described in Materials and Methods. (B) Complementation of a S. Typhimurium ∆spaO mutant (expressing the M45-tagged effector protein SopB) by the plasmid pSB4545 (pMut). Whole cell lysates (WCL) and culture supernatant proteins (Sup) were separated in a 10% SDS-PAGE gel and blotted against M45 tag to detect SopB. Figure E. Unique point mutations identified by error prone PCR depicted on the SpaO protein sequence. The start of SpaOS is indicated in red. Figure F. Example of dot blots used to screen for loss-of-funct [file ppat.1007565.s001.docx]

**Supporting Information**

**Role of SpaO in the assembly of the sorting platform of a Salmonella type III secretion system**

**Maria Lara-Tejero^1*^, Zhuan Qin^1,2^, Bo Hu^3,4^, Carmen Butan^1^,Jun Liu^1,2^, and Jorge E. Galán^1^**

Department of Microbial Pathogenesis^1^ and Microbial Science Institute^2^, Yale University School of Medicine, New haven, CT, United States; Department of Microbiology and Molecular Genetics^3^ and Pathology and Laboratory Medicine^4^, McGovern Medical School, The University of Texas Health Science Center at Houston, TX, United States.

**Figure A.** (A) Quantification of OrgA, OrgB, and InvC levels in the absence of SpaO, duplicate biological experiments loaded on the same gel. (B) Quantification of SpaO levels in the absence of OrgA, OrgB or InvC. Biological triplicates loaded on the same gel. In both cases, loading levels were normalized based on the quantification of a protein whose levels were constant as observed by Coomassie Brilliant Blue (CBB) staining (gel underneath Western blot). Quantifications were performed using the Image Studio Lite software from LI-COR Biosciences.

**
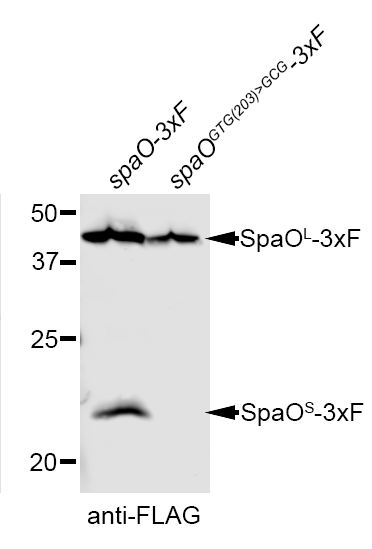
**

**Figure B.** SpaO^S^ is translated from the internal codon 203 in the *spaO* gene. (A) Whole cell lysates of either the *wild-type* strain (*spaO-3xF*) or a strain where the initiating codon for SpaOS is mutated (*spaOGTG(203)>GCG-3xF*) where run on a 15% SDS-PAGE gel and blotted against the 3xF-tag to detect SpaO^L^ and SpaO^S^.


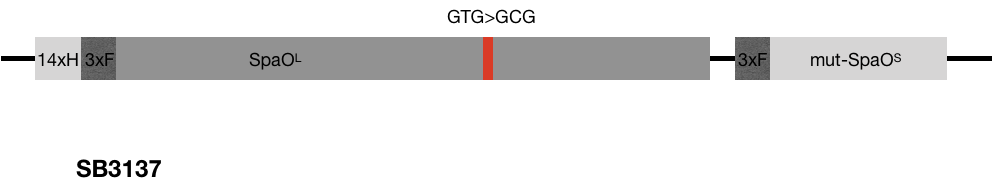


**Figure C.** Schematic representation of the *spaO* locus in the *S*. Typhimurium strain SB3137. The coding sequence of SpaO^S^ was modified without altering the protein sequence to minimize recombination between *spaO^L^* and downstream *spaO^S^*.


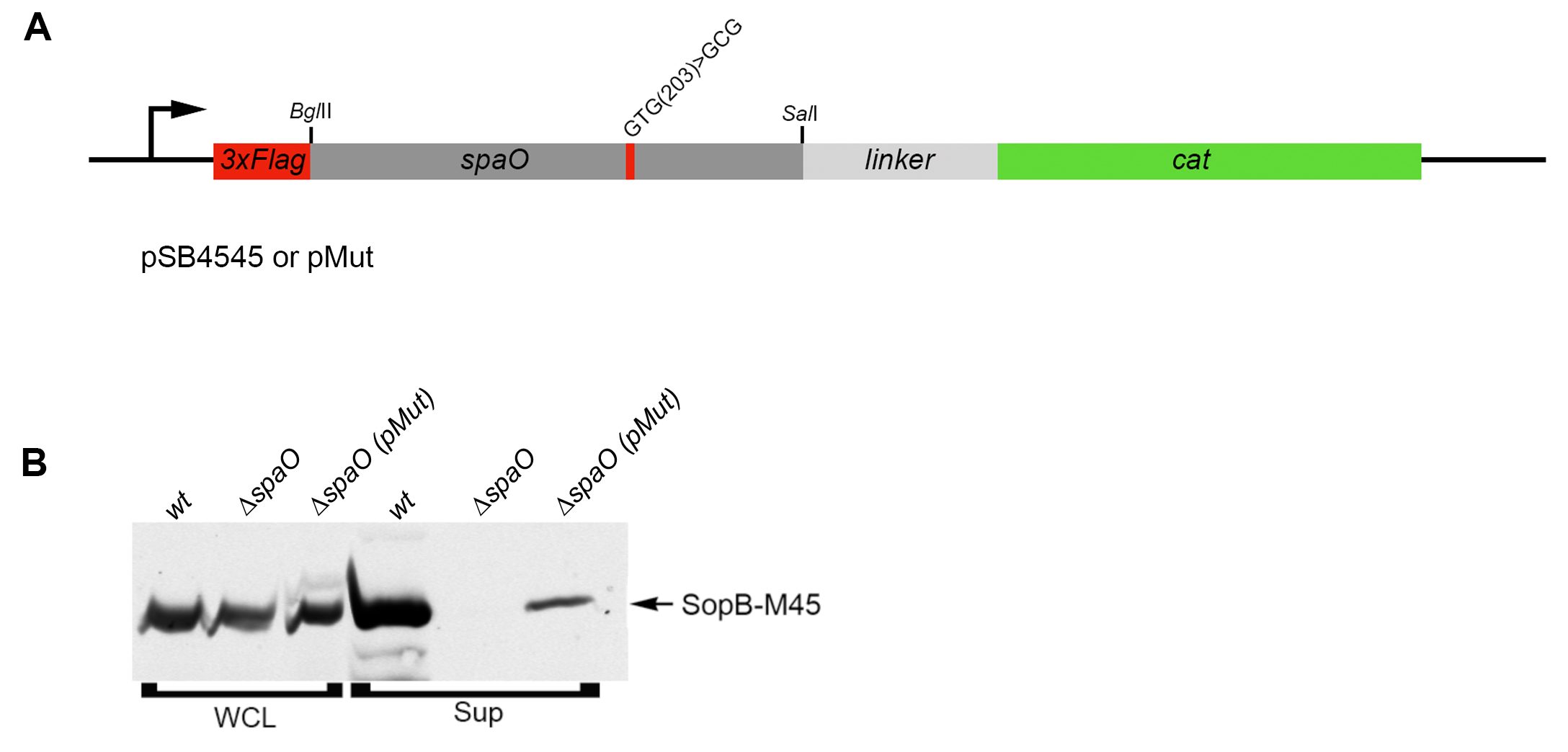


**Figure D.** (**A**) Schematic representation of plasmid pSB4545 used in the mutagenesis experiments. The plasmid expresses SpaO^L^ fused to a chloramphenicol acetyl transferase (cat) gene and carries a point mutation in *spaO* (the GTG codon at aa 203 changed to a GCG) to prevent the translation of SpaO^S^. The SpaOL open reading frame in this plasmid was mutagenized by error-prone PCR as described in Materials and Methods. (**B**) Complementation of a *S.* Typhimurium *∆spaO* mutant (expressing the M45-tagged effector protein SopB) by the plasmid pSB4545 (pMut). Whole cell lysates (WCL) and culture supernatant proteins (Sup) were separated in a 10% SDS-PAGE gel and blotted against M45 tag to detect SopB.


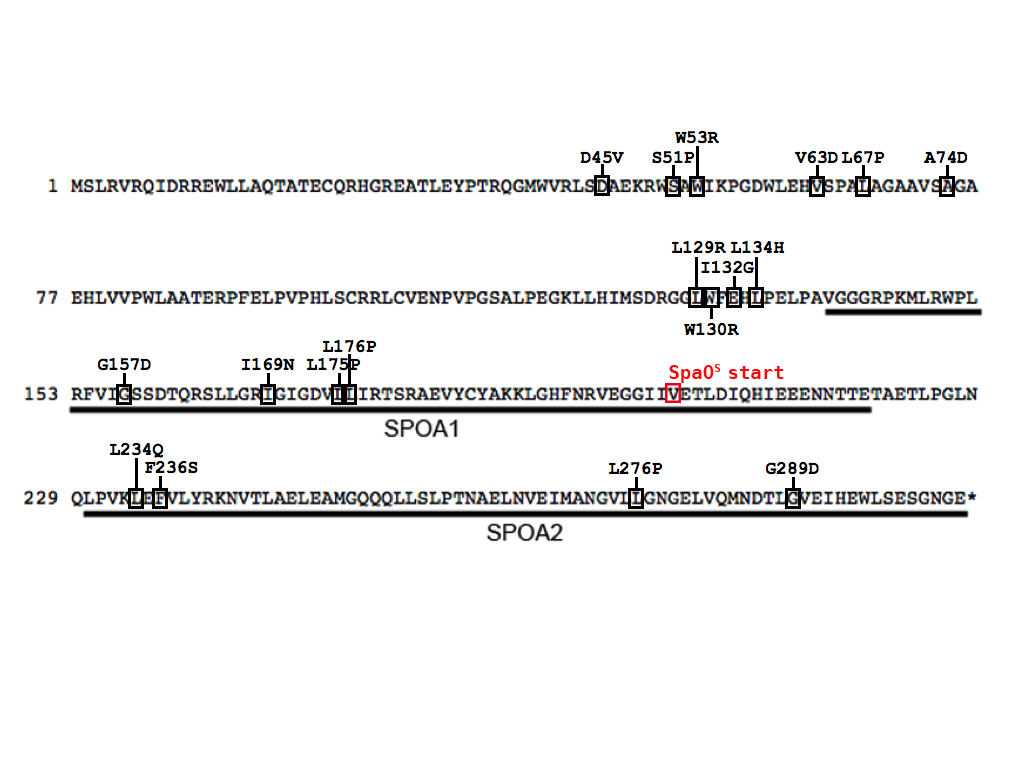


**Figure E.** Unique point mutations identified by error prone PCR depicted on the SpaO protein sequence. The start of SpaO^S^ is indicated in red.


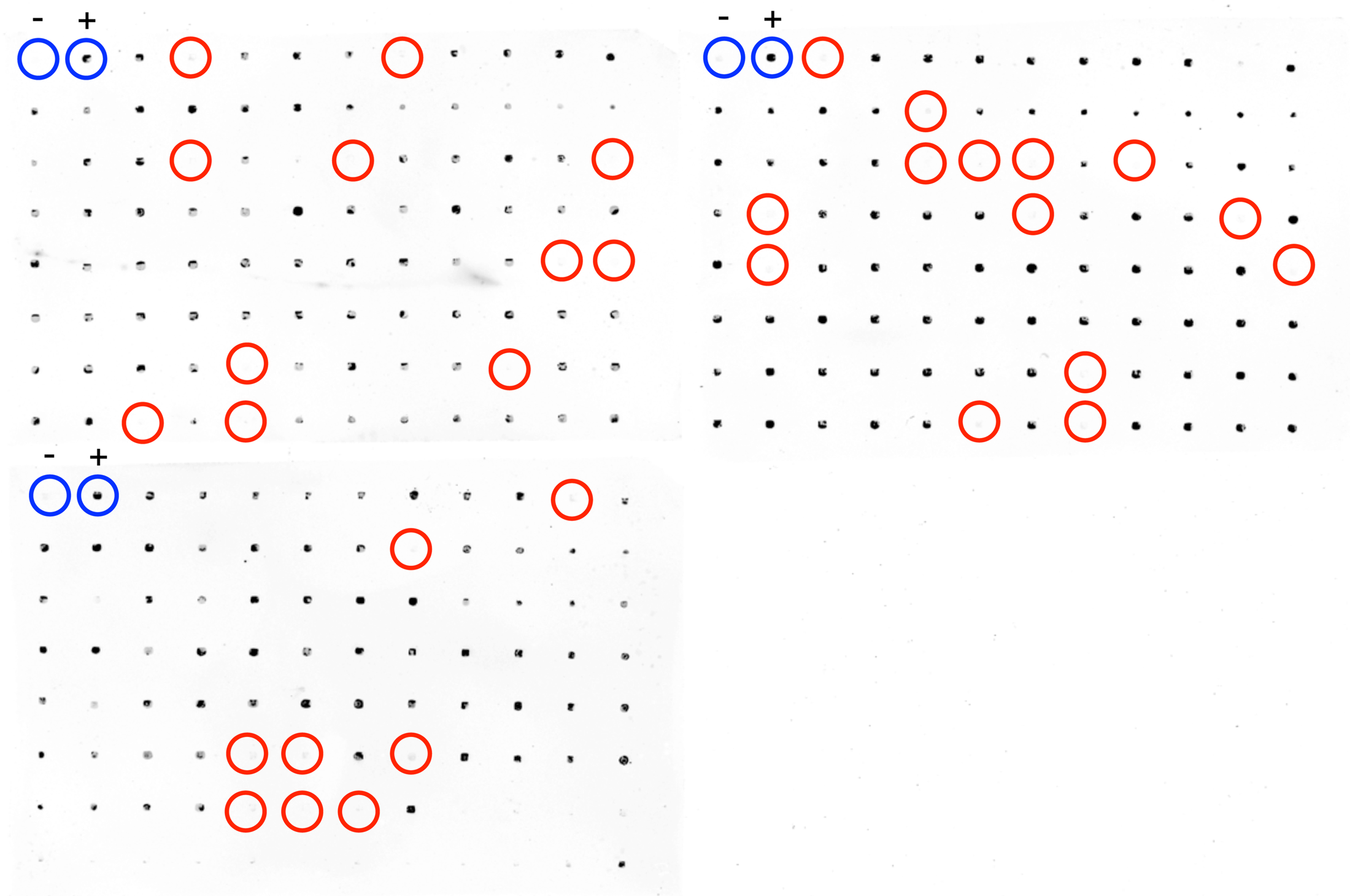


**Figure F.** Example of dot blots used to screen for loss-of-function SpaO mutants. The dot blots showed the detection of the SPI-1 T3SS effector SopB in the supernatant of *S.* Typhimurium *∆spaO* mutant complemented with the pSB4545 plasmid expressing SpaO^L^ fused to cat as positive control (+), the vector alone (-), or different SpaO^L^ mutants generated by error prone PCR as described in the Materials and Methods section. Controls are circled in blue, while putative type III secretion mutants are circled in red.


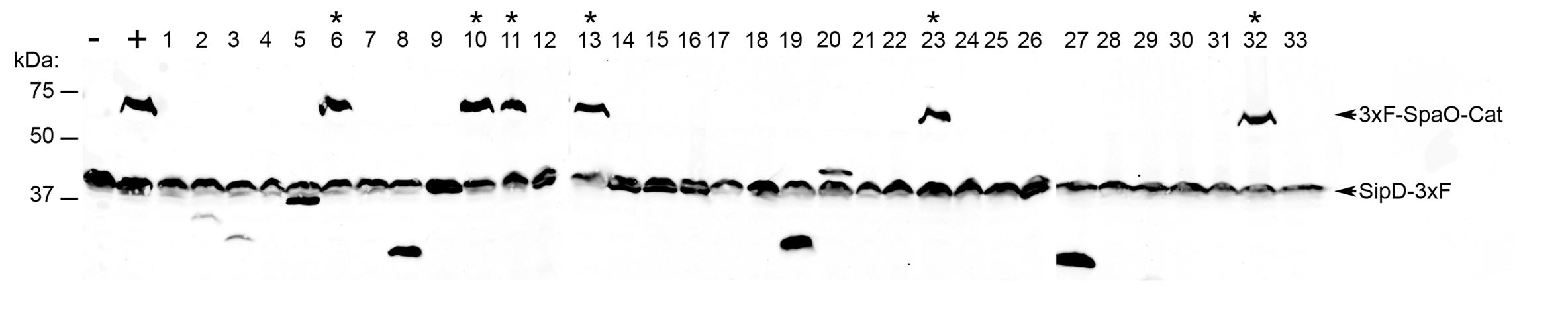


**Figure G.** An example of a western blot analysis of whole cell lysates of *S.* Typhimurium expressing different FLAG-epitope-tagged SpaO^GTG(203)>GCG^-cat fusion proteins to examine the stability of different mutants generated by error prone PCR and selected as type III secretion defective by the dot blot presented in Supplementary Figure S5. Only those mutants that expressed the full-length fusion protein (indicated by an asterisk *) were analyzed further.

**Table S1.** Strains and plasmids used in these studies.

| **Strain** | **Relevant Genotype** | **Reference** |
| --- | --- | --- |
| SB1731 | *spaO-3xF* | This study |
| SB2478 | *spaO-3xF ∆spaO^S^* | This study |
| SB1681 | *orgA-3xF* | PMID: 21292939 |
| SB2230 | *orgA-3xF ∆spaO* | This study |
| SB1682 | *orgB-3xF* | PMID: 21292939 |
| SB2231 | *orgB-3xF ∆spaO* | This study |
| SB1680 | *invC-3xF* | PMID: 21292939 |
| SB2237 | *invC-3xF ∆spaO* | This study |
| SB1741 | *3xF-spaO* | PMID: 21292939 |
| SB2126 | *3xF-spaO ∆invC* | PMID: 21292939 |
| SB2135 | *3xF-spaO ∆orgA* | PMID: 21292939 |
| SB2136 | *3xF-spaO ∆orgB* | PMID: 21292939 |
| SB2479 | *3xF-spaO^L^ ∆spaO^S^* | This study |
| SB2808 | *14xH-3xF-spaO* | This study |
| SB2796 | *14xH-3xF-spaO^L^ ∆spaO^S^* | This study |
| SB2817 | *14xH-3xF-spaO ∆orgB* | This study |
| SB2818 | *14xH-3xF-spaO^L^ ∆spaO^S^ ∆orgB* | This study |
| SB3137 | *14xH-3xF-spaO^L^ 3xF-spaO^S^ orgB-M45* | This study |
| SB2131 | *∆spaO sipA-M45* | This study |
| SB2657 | *3xF-spaO sipA-M45* | This study |
| SB2658 | *3xF-spaOD45V sipA-M45* | This study |
| SB2659 | *3xF-spaOW53R sipA-M45* | This study |
| SB2660 | *3xF-spaOV63D sipA-M45* | This study |
| SB2661 | *3xF-spaOA74D sipA-M45* | This study |
| SB2662 | *3xF-spaOE132G sipA-M45* | This study |
| SB2663 | *3xF-spaOG157D sipA-M45* | This study |
| SB2664 | *3xF-spaOL175P sipA-M45* | This study |
| SB2665 | *3xF-spaOF236S sipA-M45* | This study |
| SB2666 | *3xF-spaOL276P sipA-M45* | This study |
| SB2667 | *3xF-spaOG289D sipA-M45* | This study |
| SB2668 | *∆spaO sipA-M45 sopB-M45* | This study |
| SB2669 | *3xF-spaO sipA-M45 sopB-M45* | This study |
| SB2671 | *3xF-spaOS51P sipA-M45 sopB-M45* | This study |
| SB2674 | *3xF-spaOL67P sipA-M45 sopB-M45* | This study |
| SB2677 | *3xF-spaOL129R sipA-M45 sopB-M45* | This study |
| SB2676 | *3xF-spaOE132G sipA-M45 sopB-M45* | This study |
| SB2683 | *3xF-spaOL176P sipA-M45 sopB-M45* | This study |
| SB2684 | *3xF-spaOL234Q sipA-M45 sopB-M45* | This study |
| SB2685 | *3xF-spaOW53R ∆spaO^S^ sipA-M45 sopB-M45* | This study |
| SB2686 | *3xF-spaOE132G ∆spaO^S^ sipA-M45 sopB-M45* | This study |
| SB2687 | *3xF-spaOF236S ∆spaO^S^ sipA-M45 sopB-M45* | This study |
| SB2761 | *14xH-3xF-spaO orgB-M45* | This study |
| SB2766 | *14xH-3xF-spaOG289D orgB-M45* | This study |
| SB2779 | *14xH-3xF-spaOL67 to TAG* | This study |
| SB2790 | *14xH-3xF-spaOL234 to TAG* | This study |
| SB2794 | *14xH-3xF-spaOL276 to TAG* | This study |
| SB2797 | *14xH-3xF-spaOG289D to TAG* | This study |
| SB2798 | *14xH-3xF-spaOL67 to TAG orgB-M45* | This study |
| SB2799 | *14xH-3xF-spaOL67 to TAG invC-M45* | This study |
| SB2800 | *14xH-3xF-spaOL276 to TAG orgB-M45* | This study |
| SB2801 | *14xH-3xF-spaOL276 to TAG invC-M45* | This study |
| SB2802 | *14xH-3xF-spaOG289D to TAG orgB-M45* | This study |
| SB2803 | *14xH-3xF-spaOG289D to TAG invC-M45* | This study |
| SB2809 | *14xH-3xF-spaOL276 to TAG ∆spaO^S^* | This study |
| SB3088 | *mEos3.2-spaO^L^ ∆spaO^S^ minD::cat* | This study |
| SB3089 | *14xH-3xF-spaO^L^ mEos3.2-spaO^S^ minD::cat* | This study |
| SB3137 | *14xH-3xF-spaO^L^ 3xF-spaO^S^ orgB-M45* | This study |

| **Plasmid name** | **Description** | **Reference** |
| --- | --- | --- |
| pSB3292 | *pBAD24-hilA* | PMID: 21292939 |
| pSB890 | *R6Kori sacB tetR* | PMID: 7997169 |
| pSB4545 | *pMut, mutagenesis plasmid* | This study (see Fig S3) |
| pSB5199 | *pUC-IDT-3xFmutspaO^S^* | Synthesize for this study by IDT |

**Table S2.** Data collected for the tomographic reconstruction of the *Salmonella* injectisome.

|  | Strain |  | tomo | particle |
| --- | --- | --- | --- | --- |
| 1 | SB1780 minD::cat (wild-type strain) | WT | 1470 | 5274 |
| 2 | SB3088: PAFP-spaOV203A  minD::cat | PAFP-spaOV203A | 327 | 1829 |
| 3 | SB3089: SpaOV203 PAFP-SpaOS minD::cat | SpaOV203 PAFP-SpaOS | 455 | 1815 |
| 4 | SB3049: *SpaO^L67P^* minD::cat | *SpaO^L67P^* | 200 | 775 |

**Table S3.** Mutagenesis results summary

| **Total number of mutants obtained** | 47 |  |  |
| --- | --- | --- | --- |
| **Mutants with a single mutation** | 23 |  |  |
| **Mutants with two mutations** | 17 |  |  |
| **Mutants with three mutations** | 6 |  |  |
| **One mutant had a duplication and was discarded** |  |  |  |
|  |  |  |  |
|  |  |  |  |
| **Single mutants** | **Changed to** | **# of times picked up** | **Comments** |
| **Asp45** | Val | 1 |  |
| **Ser51** | Pro | 1 |  |
| **Trp53** | Arg | 1 |  |
| **Val63** | Asp | 1 |  |
| **Leu67** | Pro when single mutation, and to Gln in combination with other mutations | 4 | One single, 2 times in combination with Asn215 to Ile, and one time in combination with Glu219 to Lys and Lys241 to Glu |
| **Ala74** | Asp | 2 | Most likely siblings |
| **Leu129** | Arg when single mutation, and to Pro in combination with Ile156 | 2 | One single with 2 silent mutations, the other in combination with Ile156 to Val |
| **Trp130** | Arg when single mutation, and to Gly in combination with Asp207 to Val | 3 | One single, 1 single but in combination with a silent mutation, 1 in combination with Asp207 to Val |
| **Glu132** | Gly | 1 |  |
| **Leu134** | His | 1 |  |
| **Gly157** | Asp | 1 |  |
| **Ile169** | Asn | 2 | In one of them was in combination with Ile202 to Thr |
| **Leu175** | Pro | 2 | One single, another in combination with Asn285 to Ser |
| **Leu176** | Pro | 1 |  |
| **Leu234** | Gln when single mutation, and to Pro in combination with Asp9 to Glu and Leu165 to Ser | 2 | One single, another in combination with Asp9 to Glu and Leu165 to Ser |
| **Phe236** | Ser | 3 | Most likely siblings |
| **Leu276** | Pro | 1 |  |
| **Gly289** | Asp | 4 | Two times single, one time in combination with Ser99, one time in combination with Glu198 to Gly |
|  |  |  |  |
| **Double mutants** |  |  |  |
| **Leu3 to Ser + Val184 to Asp** |  | 1 |  |
| **His25 to Asn + Ser64 to Pro** |  | 1 | His25 was also mutated in combination with Leu120 and Leu245 |
| **Arg11 to His + Leu120 to Ser** |  | 1 | Leu120 was mutated two additional times in combination with other mutations |
| **Trp50 to Gly + Val72 to Pro** |  | 1 | Ser51, next to Trp50 was mutated as a single mutation, Ala 74, close to Val72 was also mutated as a single mutation |
| **Leu60 to Pro + Val197 to Asp** |  | 1 | Both mutations are unique |
| **Leu67 to Gln + Asn215 to Ile** |  | 2 | Leu67 was also picked up as a single mutation |
| **Ser99 to Pro + Gly289 to Asp** |  | 1 | Gly289 was picked 3 more time, two as a single mutation, another in combination with Glu198 |
| **Pro108 Thr + Val237 to Asp** |  | 1 | Phe236, next to Vale237, was mutation to Ser as a single mutation |
| **Leu120 to Ser + Ile177 to Val** |  | 1 | Leu120 was mutated two additional times in combination with other mutations |
| **Leu129 to Pro + Ile156 to Val** |  | 1 | Leu129 was also picked up as a single mutation, and Trp130 was picked up as a single mutation |
| **Trp130 to Gly + Asp207 to Val** |  | 1 | Trp130 was also picked up as a single mutation as well as Leu129 |
| **Ile169 to Asn + Ile 202 to Thr** |  | 1 | Ile169 was also picked up as a single mutation |
| **Asp173 to Glu + Lys241 to Met** |  | 1 | Lys241 mutated to Glu in combination with two other mutations Leu67 to Gln and Glu219 to Lys |
| **Leu175 to Pro + Asn285 to Ser** |  | 1 | Asn285 was also picked up changed to Tyr in combination with Pro260; Leu175 was picked up by itself as a single mutation |
| **Glu198 to Gly + Gly289 to Asp** |  | 1 | Gly289 was picked 3 more time, two as a single mutation, another in combination with Ser99 |
| **Pro260 to Leu + Asn285 to Tyr** |  | 1 | Asn285 was also picked up changed to Ser in combination with Leu175 |
|  |  |  |  |
| **Triple mutants** |  |  |  |
| **Asp9 to Glu + Leu165 to Ser + Leu234 to Pro** |  | 1 | Leu234 was also mutated to Gln as a single mutation |
| **His25 to Leu + Leu120 to Val + Leu245 to Arg** |  | 1 | His25 was also mutated in combination with Ser64, and Leu120 was mutated in combination with Arg11, and also in combination with Ile177 |
| **Leu67 to Gln + Glu219 to Lys + Lys241 to Glu** |  | 1 | Leu67 was picked up as a single mutation, Lys241 was picked up in combination with Asp173 in a double mutant |
| **Pro56 to His + Arg102 to Leu + Leu288 to Ile** |  | 1 | There are some single mutations closed to some of these mutations |
| **Phe91 to Leu + Ser124 to Arg + Thr217 to Ser** |  | 1 |  |
| **Pro151 to Leu + Ile156 to Asn + Gln283 to His** |  | 1 | Ile156 was also mutated in combination with Leu129, and also Gly157 was mutated as a single mutation |
